# Medicine from Ancient Greece to Soviet Russia

**Published:** 1941

**Authors:** B. Farrington

**Affiliations:** Professor of Classics, University College, Swansea


// The Bristol
Medico-Chirurgical Journal

"*Scire est nescire, nisi id me*
*Scire alius sciret.*"

AUTUMN, 1941.

### MEDICINE FROM ANCIENT GREECE TO SOVIET RUSSIA

BY

Professor B. Farrington, M.A.

*Professor of Classics, University College, Swansea.*

Being the substance of an address delivered to the Bristol Branch of the Society for Cultural Relations on 16th July, 1941.

For the purpose of this address the term medicine is used in the sense given to it by the distinguished American doctor, Le Roy Crummer, when he said that medicine meant to him not the discoveries of the great men, Vesalius, Harvey, Jenner, and the rest, but the kind of medical attention available to the common man down the centuries.[1]

I know that I am addressing a *Society for Cultural Relations*, and that I may appear to be straining the word culture in applying it to medicine regarded not simply as an art or a science, but primarily from the sociological point of view as the application of the art of medicine to the people as a whole. But this extension of the meaning of the word culture is excusable, indeed necessary, in the present instance, as the distinguishing character of medicine in the U.S.S.R. is its emphasis on the sociological side.

An historical approach to the subject, justifiable under any circumstances, is also peculiarly appropriate to medicine in the

E

U.S.S.R. ; for medicine there, like every other human activity, is, in accordance with the prevailing philosophy, regarded as a stage in an historical process. And from this point of view Soviet medical practice claims to be the culmination of the tradition of Western European medicine.

It is worth stressing this point. The word oriental has, perhaps, been too often employed in reference to the new Russia. This is unfortunate, as it creates the impression that the civilization of the U.S.S.R. is something alien to us. But, in fact, what the new Russia has done for public health has been built upon foundations laid in Western Europe, and, if it be admitted that Russia has something to teach us now in the matter of the prevention of disease, Russian practice is assuredly much indebted to the pioneering efforts of many men in various countries of Western Europe. I might mention such a man as Johann Peter Frank, the first in the world to enunciate clearly the idea that the conception of government was incomplete until it was made to include a system of medical police : or Lemuel Shattuck, the great pioneer of public health reform in the New World : or Louis Pasteur, to choose one out of the many scientists whose researches have contributed to make possible the realization of the idea of preventive medicine. Incidentally it may be observed that each of these men was as truly a son of the people as Voroshilov or Timoshenko.

Nor are English names wanting to swell the list. The Red Army and the Red Navy, like the British Army and the British Navy, owe a heavy debt to the tradition of military and naval health services of which such Englishmen as Pringle and Lind were pioneers ; while the names of Sir Edwin Chadwick and of Sir John Simon (I refer here not to the twentieth-century politician but to the nineteenth-century author of *English Sanitary Institutions*) are necessary to complete even the shortest list of those who have led the world in building up the theory and the practice of public health services.

But the movement, of which these men were pioneers, arose out of a situation as old as civilization itself. At least as far back as the fifth century B.C. in Greece we find clearly defined the two elements of the problem we are considering, that is to say, the existence on the one hand of a scientific medicine, and on the other hand the difficulty of its application to the needs of society as a whole.

Hippocratic medicine, while based on no more than an elementary knowledge of anatomy and physiology, was, as is universally admitted, thoroughly scientific in outlook. It had a conception of health and disease to which, even in quite modern times, the attention of the medical profession has been recalled, with beneficial effects on practice. The Hippocratic physician regarded life as a principle of activity which manifested itself by maintaining an organism in a certain environment. Perfect health, which exists only ideally, implies a perfect balance between organism and environment. Sickness is an effort to restore equilibrium. Health and sickness are thus two states tending to the same end, the preservation of the individual. Symptoms are cries of Nature to reveal to us the state of the body. It is the physician's business to learn to interpret these, and to decide (*a*) whether Nature is strong enough to triumph, when the physician essays merely to help her by prescribing rest, a purge, and light food ; (*b*) whether Nature is going to be beaten, when the physician must be prepared to apply more vigorous and positive measures ; and (*c*) whether Nature's own curative efforts may not be so violent that they need to be controlled and moderated by the physician's prudent care.[2]

If we add to the elementary but very intelligent medical practice based on these principles an admirable technique of surgical treatment of wounds and dislocations, we find that the Hippocratic physician had at his disposal a really serviceable art for the amelioration of the sufferings of mankind. But if we turn now to the sociological problem of the application of this knowledge and this skill to the population as a whole, the picture is not so satisfactory.

In his *Laws* Plato gives us the following glimpse of the real position of medical practice in fourth-century Greece :

> The sick in our cities are of two classes, slaves and free. Slaves are for the most part attended by slaves, who run round visiting them or wait for them in their consulting rooms. There is no discussion between patient and doctor about the particulars of each case, but, with an air of being fully informed, the doctor prescribes some empirical remedy, like an unconstitutional monarch whose will must not be questioned, and skips off to attend on the next of the sick slaves, thus relieving his master (that is, the free doctor) of the care of such patients. But free men are, as a rule, attended by a free doctor. He makes a thorough examination into the course of the disease from the beginning and its nature, takes the sick man and his friends into consultation, learns from the sick man as well as teaching him, and endeavours by gentle persuasion to lead him along the path to a full recovery.[3]

From this passage it is evident that the slaves, an increasing element in the population, were excluded from the full benefits of the medical science of the day. May it then be assumed that the best medical attention was promptly available for all free men ? Surely not. Such ideal conditions do not yet exist in our own democratic society, of which Le Roy Crummer has made the pregnant criticism : " Only the rich go to a doctor at once." That in ancient

Greece there was a difference between the medical attention available for the rich and for the poor seems proved by another passage from Plato.

In the *Republic* Socrates and Glaucon discuss the sociological aspect of medical practice in their day.[4]

" Asclepius," says Socrates, " knew that in well-ordered states every man has an occupation to which he must attend, and has therefore no leisure to spend in prolonged illness. This we remark in the case of the artisan, but, oddly enough, we fail to apply the same rule to the rich."

" How do you mean ? " said Glaucon.

" I mean this. When a carpenter is ill he asks the physician for a rough and ready cure. An emetic or a purge or a cautery or the knife, these are his remedies. But if some one prescribes for him a course of dietetics, or tells him to wrap his head up and keep it warm, he replies at once that he has no time to be ill, and that he sees no good in a life that is spent in nursing his disease to the neglect of his customary employment. He therefore bids good-bye to this sort of physician, resumes his ordinary habits, and either gets well, lives, and does his business, or, if his constitution fails, dies and is rid of his troubles."

" Yes," said Glaucon, " that's the proper sort of medicine for a man in his state of life."

From these observations of Plato, which are indeed in line with the other evidence available to us, we may safely conclude that Greek medicine did not develop as a health service for the working population, whether slave or free, but on the contrary tended rather, as Plato complains, to pander to the comfort of a leisured class. Nor did the general character of ancient medicine undergo any sensible improvement from this point of view when Greece was absorbed in the empire of Rome. It is true that Roman civilization did contribute something. Among the public utilities provided by Rome itself and by other municipalities throughout the empire, the care for the water supply, and the provision of public baths, were an important contribution to public health. But this is perhaps the only contribution of Rome to the care of the health of her population which does not painfully suggest also the vices of her regime.

Thus, the career of Asclepiades, the man who, in the first century B.C., introduced Greek medicine into Rome, reminds us of the ever-widening gap between rich and poor. Professionally he was immensely successful, but he figures in history not as a philanthropist, but as " the type of the fashionable West-end physician " [5] ; while the position of his great critic, Galen, three hundred years later, who was surgeon to the gladiators at Pergamos, is further illustration of the crazy structure of society in the Roman Empire. For, granted that it is true, in a certain sense, that his salary represented state expenditure on a health service, it was, of course, no more than a trivial item in the enormous bill for the provision of public entertainments of the most brutal character. Properly considered, Galen's professional position was one of great irony. A devoted adherent of the Hippocratic school, he had as part of the furniture of his mind the Hippocratic maxim : *Where the love of mankind is, there is the love of the Art.* But the state of society was such that he could only exercise the healing art by giving surgical attention to slaves whose duty was to kill one another for the entertainment of their medically-neglected masters.

This rudimentary notion of a state medical service which we find in connection with the gladiatorial schools had an analogue in the care given to the predial slaves on the great estates. During the period of territorial expansion of the empire, when slaves, one of the richest spoils of conquest, were cheap, medical attention for them was unknown. But when the limits of the empire had become fixed, and slaves had rather to be bred than captured, their health became a matter of concern and a sort of rough hospital service developed in connection with the *ergastula*, or slave-barracks, on the great estates. Finally, in any attempt to assess the factors which, in pagan society, led to the gradual formation of the ideal of a public health service, the organization of a medical service in connection with the army must not be overlooked.

With the triumph of Christianity an improvement begins, very noticeable in sentiment and not negligible in practice. In one of its aspects Christianity represents an upsurge of the depressed elements in ancient pagan society. Its appeal was to the weary and heavy-laden, and among them were specifically included the halt, the maim, the blind. The Founder of Christianity was *par excellence* a healer. To minister to the sick was to minister to God. A reorientation of the attitude of society to the sick man began. The fourth century is claimed as an era of great social advance. "Side by side with the strictly religious houses," a distinguished authority informs us, "there sprang up innumerable charitable institutions—orphanotrophia, ptochotrophia, nosocomia, gerontocomia, brephotrophia—intended to relieve the wants of every class and every age and not merely those of citizens, as had been the case in heathen Rome and Athens. Not the least of the debts which the world owes to fourth-century Christianity is the invention of open hospitals." [6] A consequence of this great movement is the fact that the origin of many of our hospitals is to be found in monastic provision for care of the sick and indigent.

But while Christianity may claim to have initiated a new social attitude towards the sick and the suffering, it is still a far cry from this to any comprehensive system of organized care for the physical well-being of the labouring masses as a whole. Christian charity was a work leading to salvation, undertaken more in the interest of the eternal well-being of the doer than of the temporal well-being of the patient. The secular ideal of public health is a modern thing, and, like modern humanitarianism in general, had its rise in the eighteenth century.

It is, at any rate, at the beginning of the eighteenth century that we find for the first time in history a clear enunciation of the view that medicine has a special duty to perform in safeguarding the health of the working classes. Bernardini Ramazzini was the prophet of the new idea. 1713 was the year which saw the completion of his arduous researches and the publication in its final form of his great work, *De Morbis Artificum*, or *On the Diseases of Workers*.[7] In his *Dedication* to this work the author tells us that he "thought it would be of advantage to the republic of mankind if he did what no man before him had done, that is to say, subjected the diseases of workers to special examination and suggested remedies for them." He was aware both of the novelty and the importance of the attempt, and he acquitted himself of his self-imposed task with a conscientious thoroughness. Moreover, a witty humanity in his presentation of his material imparts to his book a literary as well as a scientific value. The *De Morbis Artificum* deserves a high place in the history of humanism.

His *Preface* makes clear that he envisages the problem of disease from quite a new angle. He sees disease no longer as an accidental misfortune interfering with a man's work, as it had appeared to Plato, but as something occasioned by his work, directly conditioned and determined by the character of the work. This point of view makes of disease a directly sociological phenomenon and invests society with new responsibilities in face of it. I quote his own words from the *Preface* :—

"Necessity is the mother of all arts, liberal as well as mechanical, and they are no mean blessing. But neither are they an unmixed blessing. For it must be confessed that some arts are the cause of grave injury to their practitioners. Many an artisan has looked to his craft as a means to support life and raise a family, but all he has got from it is some deadly disease, so that he has departed this life cursing the craft to which he had applied himself."

Accordingly, " medicine, like jurisprudence, should make a contribution to the well-being of the workers, and see to it that, as far as possible, a man should exercise his trade without injury."

Hippocratic medicine, as we have seen, had been careful to consider the organism in its environment, but the environment, for the Hippocratic doctor, had been merely the *natural* environment— the water, the wind, the soil, the seasons, with a glance also at the political constitution and national customs.[8] With Ramazzini, for the first time in history, the social environment takes its proper place in the picture.

" When a doctor visits a worker's home he should be content to sit on a three-legged stool, if there isn't a gilded chair, and take time for his examination. And to the questions recommended by Hippocrates ' What sort of pains have you got ? What caused them ? How many days have you been ill ? Are your bowels working ? What is the state of your appetite ? ' he must add : ' What is the nature of your occupation ? ' "

Ramazzini himself narrates in moving style the circumstance which suggested to him the enquiry which was to be the chief work of his life.

" In our town "—he lived in Modena—" which is pretty populous for its size, and which for that reason has tall houses closely packed together, it is the custom that the drains which run in different directions through the streets should be cleaned out in each house once in every three years. When that work was going forward in our house on one occasion, I observed one of the labourers making extraordinary exertions to get through with his task. I pitied him on account of the cruel nature of the job, and asked him why he worked so hard and did not rather take it gently as so to avoid exhausting himself by his excessive exertions. The poor fellow lifted his eyes up out of the pit and fixed them upon me : ' Nobody,' he said, ' who has not tried it can imagine what it costs to spend more than four hours in this place. It's as good as going blind.' When he came out of the sewer, I examined his eyes carefully and found them blood-shot and clouded. I asked him whether the cleaners of privies had any regular cure for this trouble. ' None,' he said, ' except to go home at once, as I shall do now, shut themselves in a dark room and stay there till next day, bathing their eyes from time to time in warm water. That helps the pain.' "

His practical and unsentimental reaction to this experience was to examine in person into the conditions of work, and describe the occupational diseases, of every type of worker in his neighbourhood. The following is the list : Metal-miners, gilders, healers by inunction, chemists, potters, tinsmiths, glass-workers and mirror-makers, painters, sulphur-workers, blacksmiths, workers with gypsum and lime, apothecaries, cleaners of privies and cess-pits, fullers, oil-pressers, tanners, cheese-makers and other workers at dirty trades, tobacco-workers, corpse-bearers, midwives, wet-nurses, vintners and brewers, millers and bakers, starch-makers, sifters and measurers of grain, stone-cutters, laundresses, workers with flax, hemp and silk, bathmen, salt-makers, workers who work standing, sedentary workers, " Jews " (that is to say, old-clothes-men), runners, grooms, porters, athletes, those who strain their eyes over fine work, voice-trainers, singers, farmers, fishermen, printers, scribes and notaries, confectioners, wood-workers, grinders of razors and lancets, brick-makers, well-diggers, sailors and rowers, hunters, soap-makers. And so that nothing, if possible, should be left out, there are special discussions of camp-diseases, diseases of learned men, and health protection of nuns.

It is a social document of extraordinary richness, written plainly but with point and skill. We note the author's observation that he had "never seen an old corpse-bearer." We are shown the makers of mirrors examining their ravaged faces in the mirrors they themselves had made and cursing their choice of a trade. We see the peasants from the Campagna flocking into the hospitals at Rome, there to fall victims to the hordes of medical students with their ignorant zeal for blood-letting and purging. We learn to understand the irregularities and the hardships of the fisherman's life. And we are taught to appreciate the complexity of the problem, and its manifold social implications, in the discussion, for instance, of the brick-makers. These men, drawn from the peasant class, suffer much from fevers. The doctors, who "know nothing of the mode of life of these workers who are exhausted and prostrated by unceasing toil," prescribe the usual remedies, purging and venesection, which they have found beneficial with their idle and overfed patients among the well-to-do. But that is not the only obstacle to a sensible treatment of these poor men. "For these wretched workmen the best remedy would be a fresh-water bath at the earliest stage when they begin to have fever, for their bodies are rough and dry with dirt, and by moistening the skin and opening the pores the fever heat would be given an outlet. But nowadays the habit of taking baths has died out, though in the remote past it was well understood by doctors. At Rome of old they were in general use, and when the workmen had been at work all day they would go in the evening to the public baths, and for a trifling sum were washed clean. . . . But since the Christian religion is more concerned for the health of the soul than for that of the body, it has allowed this habit of taking baths to fall gradually into disuse."

With Ramazzini in the eighteenth century, although the serious battle for public health still lay in the future, in theory at least the sociological aspect of ill-health was clearly realized. And it is this aspect which, in our day, has been raised into the very forefront of the picture in that wisest of wise books on the art of medicine—I mean Dr. Sigerist's *Introduction to Medicine*. Here, in the chapter on *The Sick Man*, we are invited to ponder on the meaning of the words, *to be unwell*, and advised that we must put in the first place the sociological aspect of the question.

There can be no doubt, Sigerist writes, that the sick man occupies a quite special position in society. The task of the doctor does not consist in an anatomical restoration of the patient, that is to say, in treating him until all his organs, in their structure and function, are restored to exactly the same state they were in before his illness. However desirable may be this *restitutio ad integrum*, the doctor may consider his task complete when he has succeeded in snatching the patient out of his special situation, and reincorporating him as a serviceable member in society. A scar, a disfigurement, the lack of a vermiform appendix, have no importance whatever so long as they do not interfere with the capacity of the individual to work and enjoy life.

And again, towards the end of his book, he asks : " When is a treatment finished ? When is the patient cured ? " And he answers his question thus :—

" The decisive factor is of a functional, nay, of a sociological nature. A sick man is cured, our mission is accomplished, only when the individual is again fully equal to the demands which life imposes upon him, when he is re-integrated with society, whether in his old situation, or, if that is no longer possible, in a new one."

Then, with characteristic honesty, Sigerist adds : " It is not our business to find the man a new post. Here economic considerations come into play."

" It is not our business to find the man a new post." But whose business is it ? And can medicine itself avoid frustration unless it, like the sick man, is re-integrated with society ? To these questions, which are simply a repetition of the problem we undertook to consider in this address, namely, the application of the best available medical attention to the whole of a given population, Dr. Sigerist now tells us that he has found the answer, and found it in the U.S.S.R.

Before Dr. Sigerist undertook to investigate conditions in the U.S.S.R., English readers had at command a variety of sources of information of which the most accessible and the most authoritative was probably Semashko's *Health Protection in the U.S.S.R.* (Gollancz, 1934). Here it was emphasized that the basis of Soviet health protection lay in the new system of society in which unemployment had been abolished, illiteracy liquidated, and health protection organized in close connection with the productive activities of society. The protection of health, like the exploitation of productive resources, had become the concern of the central government ; health protection was co-ordinated with the general economic plan characteristic of the Soviet system, and was operated through a net-work of local agencies which in the last resort drew in every member of the public to active participation. " The health service plans are discussed at the factory meetings to see whether they correspond to the interests of the population." Intelligent participation of the layman was possible because, in addition to the appointment of duly qualified experts, provision for public health included such matters as housing conditions, public utilities (water-supply, drainage, baths, laundry, etc.), conditions of work (hours, holidays, accidents), communal feeding, children's crèches, sanatoria, health-resorts, and so forth. Semashko had been the first Commissar for Public Health and had held his post for twelve years. There was, therefore, every reason to respect his authority. Nevertheless, so large were the claims, and so important their bearing on the future of society if true, that it could not but be matter for profound satisfaction when so eminent an outside authority as Dr. Sigerist undertook to make an independent investigation.

In order to estimate the worth of his opinion it is necessary to recall in a few words the career of Dr. Sigerist. It was while he was Professor at the University of Leipzig and Director of the Institute of the History of Medicine that he wrote his famous *Introduction*, which, translated into the chief languages of Europe, holds a unique place in the medical literature of the present day. It is the peculiarity of this book that it combines an historical approach with a masterly survey of contemporary conditions, and, by the intersection of these two methods, throws, as it were, a double illumination on the field it surveys. After his triumph in Europe, Dr. Sigerist undertook the description of the American medical scene, secured an equal triumph there, and became the William H. Welch Professor of the History of Medicine at the Johns Hopkins University, Baltimore. There was still a third world to investigate and describe, the new civilization of Russia. Dr. Sigerist visited that vast territory, and returned in 1935 with a pronouncement which, coming from him, must have arrested the attention of his many admirers. "I have returned from my journey," he wrote, "with the conviction that Soviet medicine represents a form of health service adapted to the conditions of the industrial civilization of our days, and that what is happening at this moment in the U.S.S.R. is the beginning of a new period in the history of medicine."[9] Two years later he published in London his comprehensive and documented study justifying these strong words.[10] From this book, which runs to near 400 pages, I can do no more by way of conclusion to this address than quote a few sentences.

Here are the quotations :

"We know what should be done in medicine. We all know that slums breed tuberculosis, that unemployment leads to prostitution and to the spread of venereal diseases, that undernourishment cripples children. We all know that and yet we are helpless under the present system. Whenever we attack such a problem, we run headlong into economic barriers. . . .

" The Soviet Union has made a fresh start, is building a new type of society in which for the first time in history man is not exploited by his fellow-man. How are medical problems solved there ? What is done for the people's health ?

" This is what we want to know, what we have to know, what we cannot afford to ignore. What is happening in the Soviet Union is so tremendous, so significant for the Western as well as for the Eastern world, that we have to know about it (p. 17).

"It seems to me that the following four points represent the most characteristic features of the Soviet health system : (1) medical service is free and therefore available to all ; (2) the prevention of disease is in the foreground of all health activities ; (3) all health activities are directed by central bodies, the People's Commissariats of Health, with the result that (4) health can be planned on a large scale (p. 84).

" Another very characteristic feature of Soviet medicine is that it has done away with the traditional distinction between preventive and curative medicine. As a matter of fact the entire system is built upon the idea of prevention. Prophylaxis is in the foreground of all medical considerations. . . . It has been estimated that in the United States only one dollar in every thirty dollars spent for medical care goes for the prevention of disease. . . . As early as 1920 more than 60 per cent. of the total appropriation of the Commissariat of Health was spent on the prevention of disease. It would be very difficult to figure out exactly how much money is spent on prevention to-day, and how much on cure, because the distinction has disappeared. Every medical worker, wherever he may be situated, is working towards the goal of the prevention of disease.

" The general idea is to supervise the human being medically, in a discreet and unobstrusive way, from the moment of conception to the moment of death. Medical workers and medical institutions are placed wherever anyone, in the course of his life, is exposed to dangers. Medical supervision begins with the pregnant woman and the woman in childbirth, proceeds to the infant, the pre-school and school child, the adolescent, and finally the man and woman at work. . . .

" In such a state the health programme is one part of the great programme of the nation. The physician is the specialist who knows about disease, and he works towards the fulfilment of the general plan side by side with the other civil servants (pp. 92–94).

" I have approached this study as an historian, in the same detached manner in which I have studied developments and conditions in other countries and in other eras of history. And I have come to the conclusion that what is being done in the Soviet Union to-day is the beginning of a new period in the history of medicine. All that has been achieved so far, in five thousand years of medical history, represents but a first epoch : the period of curative medicine. Now a new era, the period of preventive medicine, has begun in the Soviet Union " (p. 326).

Sigerist's independent survey must surely stimulate medical men and not only medical men, but Kings, Presidents, Premiers, Ministers of Health, and all men in any station of life concerned for the welfare of mankind, to inquire more deeply into the ideals and achievements of the great country which has now so fortunately become our ally in the necessary task of defeating and destroying Nazism.

### REFERENCES.

[1] *A Doctor's Odyssey*, A. Gaylord Beaman, Johns Hopkins University Press, 1935, p. 284.

[2] Modern discussions of Hippocratic medicine are very numerous. I should like to direct special attention to *Hippocrate*, par Gaston Baissette, Grasset, Paris, 1931, pp. 183–186.

[3] Plato, *Laws*, 720 c.d.

[4] Plato, *Republic*, 406.

[5] *Galen on the Natural Faculties*, A. J. Brock, Heinemann, 1916, Intro., p. xiii.

[6] *Cambridge Ancient History*, Vol. I, p. 395. The unfamiliar Greek terms mean Orphan Homes, Beggars' Homes, Hospitals, Homes for the Aged, Children's Homes.

[7] *De Morbis Artificum*, Bernardini Ramazzini. Latin text of 1713 revised with translation and notes, by Wilmer Cave Wright. Chicago. 1940.

[8] See the Hippocratic treatise, *Airs, Waters, Places*.

[9] *L'inquiétude actuelle dans le monde médical*, Henry F. Sigerist. Schweizerische Medizinischen Wochenschrift 65.

[10] *Socialised Medicine in the Soviet Union.* Gollancz. 1937.

